# Association between CD47 expression, clinical characteristics and prognosis in patients with advanced non‐small cell lung cancer

**DOI:** 10.1002/cam4.2882

**Published:** 2020-02-11

**Authors:** Oscar Arrieta, Alejandro Aviles‐Salas, Mario Orozco‐Morales, Norma Hernández‐Pedro, Andrés F. Cardona, Luis Cabrera‐Miranda, Pedro Barrios‐Bernal, Giovanny Soca‐Chafre, Graciela Cruz‐Rico, María de Lourdes Peña‐Torres, Guadalupe Moncada‐Claudio, Laura‐Alejandra Ramirez‐Tirado

**Affiliations:** ^1^ Functional Unit of Thoracic Oncology and Personalized Medicine Laboratory Instituto Nacional de Cancerología (INCan) Mexico City Mexico; ^2^ Pathology Department Instituto Nacional de Cancerología (INCan) Mexico City Mexico; ^3^ Immunohistochemistry Unit Department of Pathology Instituto Nacional de Cancerología (INCan) Mexico City Mexico; ^4^ Clinical and Translational Oncology Group Thoracic Oncology Unit Clínica del Country Bogotá Colombia; ^5^ Foundation for Clinical and Applied Cancer Research – FICMAC Bogotá Colombia; ^6^ Clinical Research and Biology Systems Unit Universidad el Bosque Bogotá Colombia

**Keywords:** CD47, EGFR, immune checkpoint, lung adenocarcinoma, phagocytosis

## Abstract

**Objective:**

CD47 is an antiphagocytic molecule that contributes to tumor cell resistance in host immune surveillance. CD47 overexpression correlated with tumor progression and shorter survival in lung cancer. However, the expression and functional significance of CD47 in Non‐Small Cell Lung Cancer (NSCLC) has not been completely understood.

**Materials and Methods:**

In this retrospective study, CD47 expression was immunohistochemically examined in tumor biopsies from 169 NSCLC patients. The association of CD47 levels (H‐score) with clinicopathological characteristics and survival outcomes was evaluated.

**Results:**

CD47 protein was detected in 84% of patients with a median expression of 80% (0‐100). Tumor CD47 levels above 1% and 50% were found in 84% and 65.7% of patients, respectively. While, median CD47 staining index was 160 (0‐300). Patients were divided into two groups according to CD47 expression (high or low), using a cutoff value of 150. High CD47 expression was associated with wood smoke exposure (71.1% vs 28.9%, *P* = .013) and presence of EGFR (+) mutations (66.7% vs 33.3%, *P* = .04). Survival analysis carried out in the whole population did not show any association of CD47 expression and survival outcome. However, in patients with EGFR (+) mutations, CD47 expression was associated with higher progression‐free survival (PFS) (12.2 vs. 4.4 months, *P* = .032). When the survival analysis was performed according to CD47 levels (cut off value: 150), both, PFS and overall survival (OS) were shortened in patients with a high expression of CD47 (10.7 vs. NR, *P* = .156) and (29.2 vs. NR months *P* = .023), respectively.

**Conclusions:**

CD47 overexpression is not a prognostic factor for PFS and OS in NSCLC patients. However, the presence of EGFR mutations and high expression of CD47 were associated with shortened PFS and OS. Coexpression of these markers represents a potential biomarker and characterizes a therapeutic niche for lung cancer.

## Graphical abstract

High CD47 expression was found in NSCLC patients harboring EGFR mutation and correlated with a worsened clinical outcome based on a low progression free‐survival.

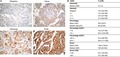

## 1 INTRODUCTION

Lung cancer (LC) remains the leading cause of cancer‐related deaths worldwide, with approximately 2.5 million new cases and 1.5 million deaths per year.^1^ Non‐Small Cell Lung Cancer (NSCLC) accounts for approximately 85% of all cases with less than 21% of overall survival (OS) rate to 5 years.^2^ Development of targeted therapy and immunotherapy has revolutionized NSCLC treatment. Molecular alterations of EGFR and ALK, and development of tyrosine kinase inhibitors (TKI´s) have improved the response rate and OS in NSCLC patients.^3^, ^4^ However, less than 20% are candidates receive TKI‐based therapy, so the prognosis for patients with advanced NSCLC remains poor.^5^, ^6^

Tumor development is a process that involves an interplay between cancer cells, normal stroma and defense system.^7^ The equilibrium between the immune system and tumor cells is disrupted during carcinogenesis, conferring to tumors the capacity to escape from host immune elimination through an immune editing process.^7^, ^8^ Incorporation of immune checkpoint inhibitors (ICI´s) against T‐lymphocyte‐associated antigen 4 (CTL‐4), programmed cell death 1 (PD‐1) and PD‐1 ligand (PDL‐1), represents an option for treatment in NSCLC patients without druggable genetic alterations.^8^ Despite the fact that patients treated with ICIs show durable responses and an increase of median OS, a portion of them do not respond and others progress during treatment.^9^

Macrophage targeting opens new possibilities for cancer immunotherapy, and tumor‐associated macrophages (TAMs) and plays a fundamental role in the maintenance of a suppressive tumor microenvironment. TAMs have emerged as potential targets of immunotherapy, because they promote activation and elimination of tumor cells through phagocytosis 10

Cluster of differentiation 47 (CD47) is a receptor ubiquitously expressed in normal cells that regulates phagocytosis.^11^ Inhibition of phagocytosis occurs when CD47 binds to signal regulatory protein alpha (SIRPα) expressed on the macrophage surface.^12^, ^13^ CD47 overexpression is associated with growth and progression in various cancer types such as non‐Hodgkin's lymphoma, gastric, colorectal, bladder, breast cancer and NSCLC.^14^, ^15^, ^16^ We have previously reported that CD47 overexpression in whole‐blood samples from NSCLC patients is associated with poor OS, and its expression on neutrophil surface prevents apoptosis and phagocytic clearance of these cells.^14^ Use of anti‐CD47 antibodies for treatment of non‐Hodgkin lymphoma, breast, bladder, and ovarian carcinomas has shown promising results.^12^, ^17^, ^18^, ^19^, ^20^ However, data regarding CD47 expression and its potential relation with clinical outcomes in lung cancer patients remain limited. In this study, we determined CD47 expression by immunohistochemistry and its relation with clinical characteristics, genetic alterations and survival outcomes.

## 2 MATERIALS AND METHODS

### 2.1 Patients and study design

This is a retrospective study; we analyzed the collected tissue biopsies, and clinical data from 169 NSCLC patients from the Instituto Nacional de Cancerología (INCan) between March 2012 and September 2016. Patients were included according to the following criteria: ≥18 years of age, high stage (IIIb or IV), histology confirmation of NSCLC, Eastern Cooperative Oncology Group Performance Status (ECOG PS) ≤2. Patients were eligible to receive platinum‐based chemotherapy or TKIs (Erlotinib or Gefitinib) according to EGFR status.

Clinical and pathological characteristics were collected from medical records. All procedures complied with the ethical standards of the Institutional Review Board as well as the Ethical Committee of INCan (011/018/ICI‐CV/683) and the Helsinki Declaration of 1975.

### 2.2 Immunohistochemistry and CD47 H‐score

Briefly, tissue sections of formalin‐fixed paraffin‐embedded (FFPE) samples (5 µm) were deparaffinized, blocked for endogenous peroxidase activity with hydrogen peroxide. Antigen retrieval was performed with immune heat‐DNA retriever citrate (cat # BSB 0023, Bio SB, Inc). Samples were washed with 1X Tris‐ buffered saline (TBS Automation Wash Buffer, 40X), and incubated with an anti‐CD47 antibody (clone: B6H12, 1:50, cat#sc‐12730; Santa Cruz Biotechnology) at room temperature for 45 minutes. This anti‐CD47 antibody is recommended by The Human Protein Atlas for the detection of CD47.^18^, ^21^ The reaction was visualized using MACH 4 universal HRP‐polymer kit (cat # M1U539, Biocare) followed by incubation with diaminobenzidine for 3 minutes. Sections were then counterstained with hematoxylin and ammonium hydroxide. Isotype‐matched IgG was used as a control for staining, and prostate tissue was used as a positive control. Each slide contained lung tissue and prostate tumor sample. A blinded examination process was performed by an independent pathologist. CD47 staining intensity and the percentage of stained cells were measured.

**Figure 1:**
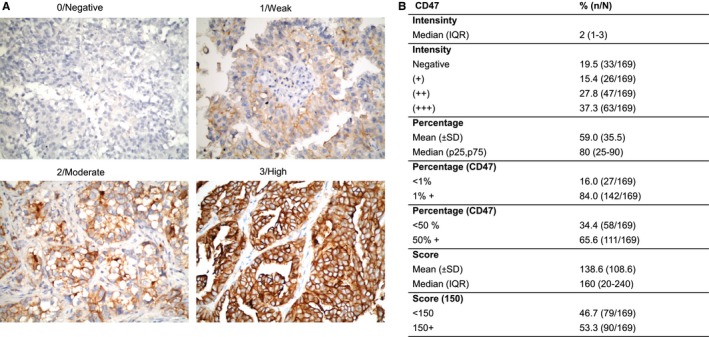
CD47 expression in NSCLC patients. A, Representative immunohistochemical staining of CD47 (brown signal) from human NSCLC biopsies showing the score system used. Magnification, x 400. Staining index of CD47. B, Median intensity, percentage of expression and score of CD47

CD47 expression index was calculated according to the method used in the EGFR FLEX trial.^19^ Tumor samples were scored according to the fraction of stained cells at each intensity. Staining intensity of the cell membrane was scored within a scale ranging from 0 to 3, and it was divided into four categories as follows: no staining, 0; weak staining, 1+ (light brown membrane staining); intermediate staining, 2+; and strong staining, 3+ (dark brown linear membrane staining). For more reliable scoring definitions, strong staining (3+) was clearly visible using a 4× objective lens, moderate staining (2+) required a 10× or 20× objective lens for clear observation, and weak staining (1+) required a 40× objective lens. Multiplication of intensity of staining and percentage of immunoreactive cells resulted in an immunoreactivity scoring system, ranging from 0 to 300 for each individual case (Figure 1A).

Additionally, PDL‐1 was evaluated by IHC using VENTANA PD‐L1 (SP263) assay.

### 2.3 EGFR mutations and ALK rearrangements

Detection of EGFR mutations was performed by Real‐Time PCR based on ARMS/Scorpions technology using the EGFR RGQ PCR Kit (cat # 870101, Qiagen). ALK rearrangement was performed using Vysis ALK Break Apart FISH Probe Kit.

### 2.4 Statistical analysis

To determine the prognostic and predictive value of CD47 expression, a cutoff point was determined. We analyzed the survival data with the specialized X‐tile software, with a minimal p‐value approach for cut off optimization.^20^

For descriptive purposes, continuous data were summarized as arithmetic means with standard deviation (SD). Data distribution was assessed using Kolmogorov‐Smirnov test. Comparison between groups was performed using Student's t test or Mann‐Whitney U‐test, depending on data distribution. Data from contingency tables were analyzed using Chi‐squared and Fisher's exact test. Overall survival (OS) and progression‐free survival (PFS) were estimated using the Kaplan‐Meier method, and comparisons among survival times were analyzed with Log‐rank test. For survival‐curve analysis, all variables were dichotomized (for age, median was used). Adjustment for potential confounders was addressed with a multivariate Cox proportional regression analysis, and hazard ratios were estimated along with their corresponding 95% confidence intervals (CI). A two‐sided *P*‐value of < .05 was considered statistically significant. Data were analyzed with the SPSS software package version 20 (SPSS, IBM, Inc).

## 3 RESULTS

### 3.1 Baseline clinical characteristics

The mean age at diagnosis was 60.9 (±12.4) years. One‐hundred and five patients were women (62.1%). Approximately 40% of patients were smokers, with a mean tobacco index of 18.4 (±19.5), and only 38 patients (22.5%) reported wood smoke exposure. Most patients presented ECOG PS < 2 at the time of diagnosis (85.2%). Adenocarcinoma was the predominant histological type, with approximately 97% of all cases, while the acinar and solid subtypes were 33.5% and 28%, respectively. One hundred fifty‐five patients (91.7%) presented with stage IV disease, while the other 14 (8.3%) presented with stage IIIB. Metastases were found in different anatomic locations, such as contralateral lung (30.3%), central nervous system (25.8%), bone (20%), and liver (17.4%). Regarding mutational status, 24.9% of patients harbored EGFR mutations, while 9.9% harbored ALK rearrangements. Also, only 4.7% of the patients had a high expression of PD‐L1 (TPS > 50%).

IHC staining (Figure 1) showed that CD47 was primarily located in the cytoplasmic membrane of tumor cells and to a lesser extent, diffusely in the cytoplasm. However, there was a significant variability in staining intensity and percentage of positively stained cells among patients. The 1% + expression percentage for CD47 was 84%. While 65.7% presented an expression percentage of 50% + (Figure 1). 80% of patients (25 to 90) presented CD47 expression inside the median ( from percentile 25 to percentile 75), and median staining index was 160.

### 3.2 Association of CD47 scores with clinical characteristics

Based on staining index scores, 169 patients were stratified into two groups: those with a low CD47 score (<150) (n = 79) and those with a high CD47 score (150+) (n = 90). Table 1 shows the baseline characteristics of NSCLC patients according to CD47 score. Higher CD47 score (150+) was related to wood smoke exposure (71.1% vs 28.9; *P* = .013) and presence of EGFR mutations (66.7% vs 33.3%; *P* = .044). No differences were found between chemotherapy (QT) or TKI treatment (Table 1).

**Table 1:** Clinical characteristics according to CD47 expression

	ALL (N = 169)	CD47 (<150)	CD47 (≥150)	*P*‐value
		(N = 79)	(N = 90)	
	% (n/N)	% (n/N)	% (n/N)	
Sex				
Male	37.9 (64/169)	43.8(28/64)	56.3(36/64)	
Female	62.1(105/169)	48.6(51/105)	51.4(54/105)	.542
Age				
Mean (±SD)	60.9 (12.4)	60.8 (11.9)	61.0 (12.8)	.911
<60 y	41.4 (70/169)	48.6(34/70)	51.4(36/70)	
≥60 y	58.6 (99/169)	45.5(45/99)	54.5(54/99)	.689
Tobacco exposure				
Mean (±SD)	18.4 (19.5)	19.1 (19.8)	18.0 (19.7)	.865
Nonsmoker	59.8 (101/169)	48.5(49/101)	51.5(52/101)	
Ever smoker	40.2 (68/169)	44.1(30/68)	55.9(38/68)	.574
WSE				
Absent	77.5 (131/162)	51.9(68/131)	48.1(63/131)	
Present	22.5 (38/169)	28.9(11/38)	71.1(27/38)	**.013**
ECOG PS				
<2	85.2 (144/169)	45.8(66/144)	54.2(78/144)	
2+	14.8 (25/169)	52.0(13/25)	48.0(12/25)	.568
Histology				
Adenocarcinoma	97.0 (164/169)	46.3(76/164)	53.7(88/164)	
Squamous	3.0 (5/169)	60.0(3/5)	40.0(2/5)	.666
Architectural grade				
High‐Moderate	59.8 (98/164)	45.9 (45/98)	54.1 (53/98)	
Low	32.2 (53/164)	41.5 (22/53)	58.5 (31/53)	
Unspecified	7.9 (13/164)	69.2 (9/13)	30.8 (4/13)	.198
Disease stage				
IIIB	8.3 (14/169)	42.9(6/14)	57.1(8/14)	
IV	91.7 (155/169)	47.1(73/155)	52.9(82/155)	.761
1^ry^ Metastatic sites				
Contralateral lung	30.3 (47/155)	53.2(25/47)	46.8(22/47)	.316
CNS	25.8 (40/155)	50.0(20/40)	50.0(20/40)	.669
Bone	20.0 (31/155)	51.6(16/31)	48.4(15/31)	.573
Liver	17.4(27/155)	55.6(15/27)	44.4(12/27)	.333
CEA				
<5 pg/mL	25.4 (43/169)	48.8(21/43)	51.2(22/43)	
≥5 pg/mL	74.6 (126/169)	46.0(58/126)	54.0(68/126)	.75
EGFR				
EGFR^wt^	75.1 (127/169)	51.2(65/127)	48.8(62/127)	
EGFR^+^	24.9 (42/169)	33.3(14/42)	66.7(28/42)	**.044**
ALK				
Absent	95.3 (161/169)	46.6(75/161)	53.4(86/161)	
Present	4.7 (8/169)	50.0(4/8)	50.0(4/8)	1
PD‐L1				
Absent	90.1 (100/111)	34.0 (34/100)	66.0 (66/100)	
Present	9.9 (11/111)	54.5 (6/11)	45.5 (5/11)	.199
QT régimen				
CBP + Paclitaxel	26.8 (34/127)	26.2 (17/65)	27.4 (17/62)	
CBP + Pemetrexed	22.8 (29/127)	23.1 (15/65)	22.6 (14/62)	
Cisplatin + Pemetrexed	41.7 (53/127)	43.1 (28/65)	40.3 (25/62)	
CBP + Gemcitabine	7.1 (9/127)	7.7 (5/65)	9.7 (6/62)	.974
TKI régimen				
Erlotinib	28.6 (12/42)	28.6 (4/14)	28.6 (8/28)	
Afatinib	19.0 (8/42)	21.4 (3/14)	17.9 (5/28)	
Gefitinib	52.4 (22/42)	50.0 (7/14)	53.6 (15/28)	.958

Abbreviations: ALK, Anaplastic Lymphoma Kinase; CBP, Carboplatin; CEA, Carcinoembryonic antigen; CNS, Central Nervous System; ECOG PS, Eastern cooperative oncology group performance status; EGFR, Epidermal Growth Factor; QT, Chemotherapy; PDL‐1, Programmed death‐ligand 1; SD, Standard Deviation; TKI, Tyrosine kinase inhibitors; WSE, Wood smoke exposure.

No differences were found in terms of CD47 intensity. However, percentage of expression and CD47 score was higher in the EGFR‐mutated patients (median 70; 15, 80 *P* = .004 and 172.1 vs 130.9, *P* = .029, respectively) (Figure S1).

### 3.3 Progression‐free survival in NSCLC patients

**Figure 2:**
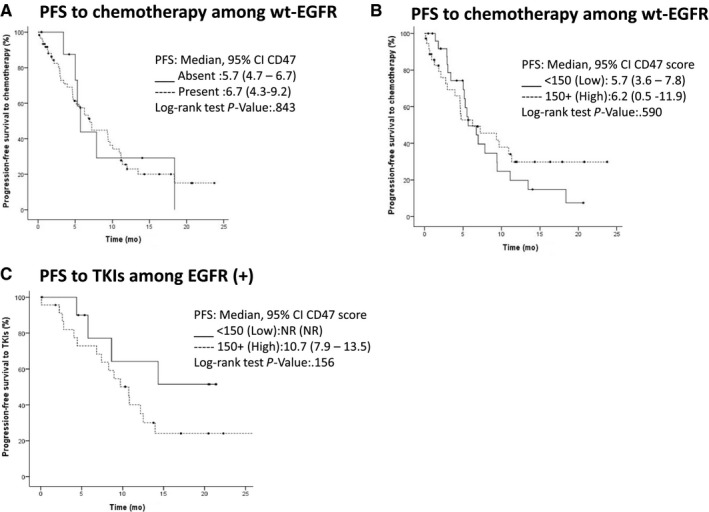
Kaplan‐Meier curves of PFS and EGFR mutational status. A and B, Kaplan‐Meier curves of PFS stratified according to EGFR‐WT and CD47 presence or expression level (cut‐off point 150). C, Curves of PFS stratified according to EGFR (+) and CD47 expression level

The median follow‐up of patients was 17.5 months (9.2‐25.9 months). Median progression‐free survival (PFS) for first‐line therapy was 8.3 months (95% CI 5.8‐10.8 months). Factors associated with better PFS were ECOG PS (ECOG < 2 vs ≥2; 9.4 months vs 3.9 months; *P* = .001), disease stage at diagnosis (IIIB vs IV; not reached vs 7.9; *P* = .034) and EGFR (+) status (10.8 vs 6.2 months, *P = *.06). Of note, no significant differences were found in PFS between high and low CD47 scores (7.9 vs 8.9, *P* = .936). Multivariate analysis showed two independent factors related to worse prognosis, ECOG PS (HR 2.8, 95%CI 1.4‐5.4; *P* = .003) and histological grade (HR 1.8, 95%CI 1.0‐3.2; *P* = .039) Table 2. CD47 expression (presence, intensity or H‐score) was not a prognostic factor associated with PFS in patients who received chemotherapy (Figure 2A,B).

**Table 2:** Univariate and multivariate analysis for PFS and OS

	Progression‐free survival				Overall survival			
	Mean, 95% CI	*P*‐value	HR (95%, CI)	*P*‐value	Mean, 95% CI	*P*‐value	HR (95%, CI)	*P*‐value
Overall	8.3 (5.8‐10.8)				25.6 (20.8‐30.4)			
Sex								
Male	10.7 (6.6‐14.9)				23.9 (17.5‐30.3)			
Female	6.7 (4.3‐9.2)	.092	1.4 (0.8‐2.5)	.243	28.3 (20.5‐36.1)	.077	0.8 (0.5‐1.4)	.558
Age								
<60 y	8.3 (5.0‐11.5)				29.9 (26.7‐33.2)			
≥60 y	8.6 (5.9‐11.3)	.219			22.1 (19.2‐25.1)	**.048**	1.5 (0.9‐2.3)	.113
Tobacco exposure								
Nonsmoker	6.9 (4.6‐9.4)				23.1 (17.6‐28.6)			
Smoker	9.7 (5.6‐13.8)	.223			26.6 (18.9‐34.3)	.676		
Wood smoke exposure								
Absent	9.4 (6.2‐12.6)				23.9 (18.3‐29.5)			
Present	5.0 (2.2‐7.8)	.087	1.4 (0.8‐2.6)	.197	26.9 (14.6‐39.2)	.549		
ECOG PS								
0‐1	9.4 (7.0‐11.8)				28.9 (26.2‐31.8)			
2+	3.9 (2.2‐5.7)	**.001**	2.8 (1.4‐5.4)	**.003**	19.4 (14.0‐24.7)	**.001**	2.4 (1.3‐4.4)	**.003**
Disease stage								
IIIB	NR (NR)				NR (NR)			
IV	7.9 (5.4‐10.3)	**.034**			23.9 (19.0 −28.8)	.365		
Histology								
Adenocarcinoma	8.6 (6.2‐11.1)				25.6 (20.8‐30.4)			
Squamous	5.3 (3.9‐6.6)	.331			18.2 (NR)	.973		
Histological grade								
High‐Moderate	9.7 (7.9‐11.6)				27.5 (21.8‐33.3)			
Low	6.7 (4.2‐9.3)	.062	1.8 (1.0‐3.2)	**.039**	17.7 (14.1‐21.3)	**.029**	1.8 (1.1‐2.8)	**.014**
Contralateral Lung metastases								
Absent	9.4 (6.6‐12.2)				23.9 (18.0‐29.8)			
Present	5.5 (4.6‐6.4)	.051	1.6 (0.9‐2.9)	.124	21.5 (12.4‐30.5)	.809		
CNS metastases								
Absent	7.9 (5.7‐10.0)				23.9 (18.7‐29.2)			
Present	9.4 (1.8‐17.0)	.693			22.1 (8.2‐36.1)	.734		
Bone metastases								
Absent	8.6 (5.6‐11.7)				23.9 (18.8‐29.1)			
Present	6.8 (1.2‐12.4)	.197			23.1 (13.6‐32.5)	.955		
CEA								
<5 pg/mL	9.7 (7.6‐11.9)				36.9 (18.5‐55.3)			
≥5 pg/mL	7.2 (5.2‐9.3)	.486			23.9 (18.6‐29.3)			
EGFR status								
EGFR^wt^	6.2 (4.1‐8.4)				20.9 (17.1‐24.7)			
EGFR^+^	10.8 (6.8‐14.9)	.06	0.7 (0.4‐1.2)	.156	39.8 (27.2‐52.3)	**.001**	0.4 (0.2‐0.8)	**.004**
CD47 score								
Absent	5.7 (4.5‐6.8)				25.6 (16.5‐34.7)			
Present	9.3 (6.9‐11.7)	.409			26.3 (20.1‐32.4)	.912		
CD47 score								
<150	7.9 (4.2‐11.5)				23.3 (17.4‐29.2)			
≥150	8.9 (5.6‐12.2)	.936			27.5 (18.9‐36.2)	.976		

Abbreviations: CEA, Carcinoembryonic antigen; CI, Confidence interval; CNS, Central Nervous System; ECOG PS, Eastern cooperative oncology group performance status; EGFR, Epidermal Growth Factor; HsssR, Hazard ratio; NR, Not reach.

### 3.4 Predictors of overall survival in NSCLC patients

**Figure 3:**
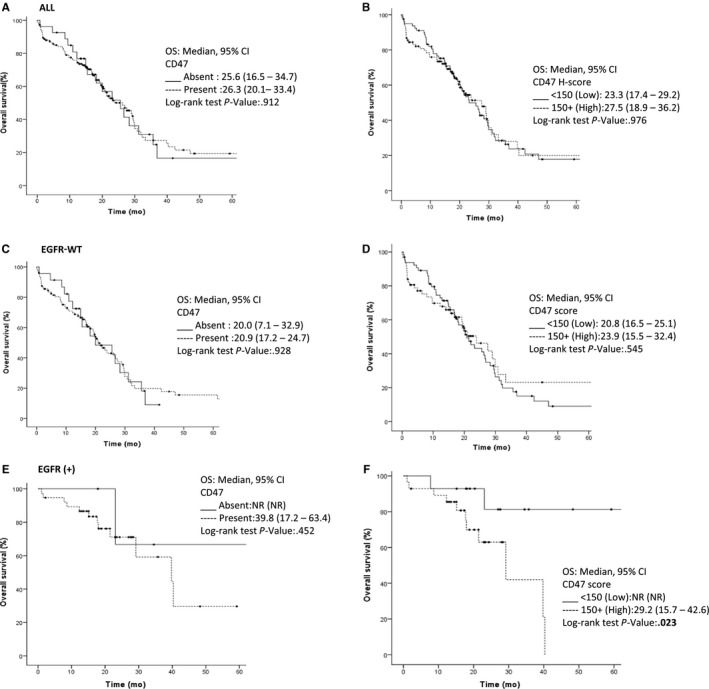
Kaplan‐Meier curves for OS in all patients (A) OS curves according to CD47 presence (B) OS curves according to CD47 levels in all patients (C) OS curves for EGFRwt in CD47 absence or presence and (D) high or low CD‐47 expression. (E) Patients with or without EGFR mutations and (F) CD47 expression higher or lower than 150. CEA, Carcinoembryonic antigen; CI, Confidence interval; CNS, Central Nervous System; ECOG PS, Eastern cooperative oncology group performance status; EGFR, Epidermal Growth Factor; NR, Not reach; NC, Not calculated; TKI, Tyrosine kinase inhibitors

Median OS for first‐line therapy was 25.6 months (95% CI 20.8‐30.4). Factors associated with better OS were age (<60 vs ≥60, 29.9 vs 22.1; *P* = .048), ECOG PS (<2 vs ≥2; 28.9 vs 19.4 months; *P* = .001), tumor differentiation grade (27.5 vs 17.7; *P* = .029), and EGFR (+) mutation status (39.8 vs 20.9 months; *P* = .001). OS was not affected by any other clinical or pathological variables such as ALK fusions, PD‐L1, or CD47 expression (Figure 3A,B; Table 2). In the multivariate analysis, ECOG PS (HR 2.4, 95%CI: 1.3‐4.4; *P* = .003) and histological grade (HR 1.8, 95%CI: 1.1‐2.8; *P* = .014) were independent factors for worse OS, while EGFR (+) mutation status was a better prognostic factor (HR 0.4, 95%CI: 02.‐0.8; *P* = .004) (Table 2).

### 3.5 Progression‐Free survival according to EGFR‐mutation status

The only factor associated with better PFS in EGFR wild‐type tumors was ECOG PS (7.2 vs 3 months, *P* = .020) (Figure 2C). Among patients harboring EGFR mutation, factors independently associated with a higher PFS were: a younger age < 60 (5.9 vs 8.9 *P* = .027), ECOG PS (12.5 vs 4.4 months *P* = .002), and pulmonary bilateral disease (12.2 vs 8.3 months, *P* = .042). Due to incomplete data to follow‐up for progression, it was not possible to determine PFS in patients with mutated EGFR (+) in the presence of CD47 (Table 3).

**Table 3:** Univariate and multivariate analysis for PFS and OS according to EGFR mutation status

	Progression‐free survival								Overall survival							
	To chemotherapy among wt‐EGFR				To TKIs among EGFR (+)				To chemotherapy among wt‐EGFR				To TKIs among EGFR (+)			
	Univariate		Multivariate		Univariate		Multivariate		Univariate		Multivariate		Univariate		Multivariate	
	Median, 95% CI	*P*‐value	Median, 95% CI	*P*‐value	Median, 95% CI	*P*‐value	Median, 95% CI	*P*‐value	Median, 95% CI	*P*‐value	Median, 95% CI	*P*‐value	Median, 95% CI	*P*‐value	Median, 95% CI	*P*‐value
Sex																
Male	9.7 (1.4‐17.9)				12.2 (7.9‐16.4)				19.5 (11.9‐27.2)				29.2 (20.2‐38.2)			
Female	5.3 (4.0‐6.7)	.102	1.6 (0.8‐3.3)	.191	7.4 (0.3‐14.5)	.682			21.5 (17.5‐25.4)	.071	0.8 (0.5‐ 1.3)	.347	NR(NR)	.603		
Age																
<60 y	5.2 (3.7‐6.6)				55.9 (NR)				28.9 (14.1‐43.9)				NR(NR)			
≥60 y	6.9 (2.7‐11.3)	.648			8.9 (5.2‐12.7)	**.027**	**2.4 (0.9**‐**6.2)**	**.071**	20.8 (18.0‐23.5)	.322			29.2 (17.6‐40.8)	.111	1.3 (0.8‐1.9)	.324
Tobacco exposure																
Nonsmoker	5.3 (2.5‐8.1)				9.7 (4.5‐14.9)				20.9 (18. 0‐23.8)				NR(NR)			
Smoker	9.4 (3.9‐14.9)	.392			NR (NR)	.128			25.6 (16.9‐34.3)	.862			40.3 (NR)	0.862		
Wood smoke exposure																
Absent	7.9 (3.9‐11.8)				10.8 (3.5‐18.1)				20.9 (17.2‐24.7)				40.3 (39.1‐41.5)			
Present	4.6 (2.1‐7.1)	.063	1.9 (1.0‐3.6)	**.047**	9.8 (5.3‐14.2)	.756			26.9 (12.8‐40.9)	.648			29.2 (20.0‐38.3)	.783		
ECOG PS																
0‐1	7.2 (3.5‐10.9)				12.5 (8.9‐16.2)				26.6 (19.2‐34.1)				40.3 (26.9‐57.8)			
2+	3.0 (2.6‐3.4)	**.02**	2.4 (1.0‐5.3)	**.039**	4.4 (0.9‐7.8)	**.002**	4.4 (1.3‐14.9)	**.019**	15.4 (9.7‐21.1)	**.001**	**2.7 (1.4‐5.3)**	**.002**	23.1 (14.9‐31.3)	.461		
Disease Stage																
IIIB	NR (4.6‐NR)				NR (NR)				NR (NR)				NR(NR)			
IV	5.7 (2.9‐11.1)	.09			10.8 (5.7‐NR)	.111			20.9 (9.7‐31.2)	.535			39.8 (21.5‐NR)	.367		
Histology																
Adenocarcinoma	6.7 (4.6‐8.9)				10.7 (6.8‐14.7)				21.5 (9.7‐31.9)				39.8 (21.5‐NR)			
Squamous	4.7 (1.3‐8.0)	.298			14.3 (NR)	.919			18.2 (5.2‐NR)	.679			NR(NR)	.543		
Histological grade																
High‐Moderate	9.4 (6.1‐12.7)				12.5 (9.2‐15.8)				23.3 (16.7‐29.8)				40.3 (23.8‐56.8)			
Low	5.5 (3.2‐7.9)	.119			8.6 (7.0‐10.3)	.386			14.9 (7.0‐22.8)	**.042**	**1.7 (1.1‐ 2.8)**	**.026**	39.8 (18.9‐60.6)	.533		
Contralateral lung metastases																
Absent	6.9 (3.7‐10.2)				12.2 (9.7‐14.6)				20.9 (17.3‐24.6)				40.3 (26.6‐54.0)			
Present	5.2 (3.9‐6.4)	.525			8.3 (4.3‐12.3)	**.042**			20.8 (5.7‐35.9)	.823			NR(NR)	.204		
CNS metastases																
Absent	6.2 (4.2‐8.2)				9.7 (6.6‐12.8)				21.5 (15.9‐27.0)				29.2 (10.8‐47.6)			
Present	5.0 (1.9‐8.1)	.596			15.4 (0.0‐40.9)	.34			16.8 (10.1‐23.4)	.667			NR(NR)	.118		
Bone metastases																
Absent	5.7 (3.9‐7.5)				10.7 (6.0‐15.4)				20.8 (16.9‐24.6)				39.8 (27.8‐51.7)			
Present	3.4 (0.0‐9.8)	.475			6.8 (2.0‐11.6)	.162			22.1 (8.8‐35.4)	.993			NR(NR)	.979		
CEA																
<5 pg/mL	9.4 (2.9‐15.8)				10.8 (7.7‐13.9)				36.9 (4.2‐69.5)				29.2 (NR)			
≥5 pg/mL	5.3 (2.9‐7.7)	.267			10.7 (3.2‐18.3)	.81			20.9 (17.2‐24.7)	.521			40.3 (18.6‐62.0)	.755		
CD47																
Absent	5.7 (4.7‐6.7)				NC				20.0 (7.1‐32.9)				NR(NR)			
Present	6.7 (4.3‐9.2)	.843			NC				20.0 (17.2 −24.7)	.928			39.8 (17.2‐63.4)	.452		
CD47 score																
<150	5.7 (3.6 −7.8)				NR (NR)				20.8 (16.5‐25.1)				NR(NR)			
≥150	6.2 (0.5‐11.9)	.59			10.7 (7.9‐13.5)	.156			23.9 (15.5‐32.4)	.545			29.2 (15.7‐42.6)	**.023**	1.3 (0.8‐2.0)	.31

Abbreviations: CEA, Carcinoembryonic antigen; CI, Confidence interval; CNS, Central Nervous System; ECOG PS, Eastern cooperative oncology group performance status; EGFR, Epidermal Growth Factor; HR: Hazard ratio; NR, Not reach.

### 3.6 Overall survival according to EGFR status

Among EGFR‐wt patients, a better ECOG PS (<2) and low tumor differentiation grade was independently associated with a better OS (26.6 months vs 15.4 months; *P* = .001 and 23.3 vs 14.9 months, *P* = .042, respectively). Once again, neither the expression nor CD47 score was associated with OS among wt patients. By contrast, among EGFR (+) patients, the only independently associated factor with a worse OS was a high expression (CD47 H‐score ≥ 150) (29.2 months vs NR, *P* = .023) (Figure 3C‐F).

## 4 DISCUSSION

Immune checkpoints serve as a regulatory signal to regulate the immune system and participate in the inhibition of growth and development of tumor cells. However, their overexpression on tumor cells avoids the recognition by T cells and macrophages, allowing the tumor escape of immune attack. It should be noted that CD47 is an immune control point that regulates phagocytic signaling. When CD47 is overexpressed, phagocytosis mediated by macrophages is suppressed, promoting tumor progression resulting in worse OS in a wide variety of tumors.^10^ In this study, we evaluated CD47 expression by IHC in NSCLC tumor cells based on H‐score from EGFR FLEX trial and found an optimal cutoff ≥ 150.^21^ Tumor cells have shown an expression level of ≥1% in 84% of patients, of which 65.5% had an expression ≥50%; CD47 was not a prognosis factor that response to treatment or a longer survival. The evaluation of CD47 in other studies is based only on its level of expression, where high CD47 expression was correlated with a worse OS related to the type of tumor, the method of detection, and the kind of analysis.^22^ In this report, no differences in PFS or OS were found according to CD47 expression (analyzed either as presence/absence or using a score value of 150). Previous reports showed that high RNA levels of CD47 was associated with worse PFS and OS in NSCLC patients.^23^ Furthermore, overexpression of CD47 was associated with tumor characteristics (from the TNM Classification of Malignant Tumors), clinical staging, lymph node metastasis and distant metastasis in NSCLC patients.^22^, ^24^ Although high CD47 expression could be a potential prognosis biomarker, more studies are necessary to determine the best cutoff for this molecule.

Interestingly, we found that high CD47 expression was correlated with the presence of EGFR mutations in 66.7% of our population study. Current evidence indicates that EGFR mutations and EGF stimulation reshape the immune microenvironment and modulate the expression of immune molecules like PD‐L1 and possibly CD47.^25^ Although there is no evidence of CD47 regulation by EGFR, different reports in vitro show that PD‐L1 can be either upregulated or downregulated depending on the activation or inhibition of EGFR‐mutant cell lines.^26^ Consistently, blocking the PD1/PDL‐1 axis in EGFR‐mutant lung tumors of mice results in better OS.^27^ This association of EGFR activating mutations, mainly exon 19 deletions and L858R with high PD‐L1 expression, has also been observed in patients with lung adenocarcinoma histology; however, subpopulations with this characteristic are less.^28^ Besides EGFR, other oncogenes like c‐Myc are able to upregulate CD47 and PD‐L1 expression, and when c‐Myc is inactivated induce a rapid downregulation of these immune checkpoints, improving immune response in mouse model tumors.^29^

On the other hand, the only factor associated with high CD47 H‐score (≥150) was wood smoke exposure (WSE). Chronic exposure to wood smoke is a common risk factor for lung cancer in Mexico and Latin American countries,^30^ WSE affects the expression profiles of genes like EGFR, SMARCB1, ATM, and KDR, and also activates signaling pathways such as PIK3CA/AKT and MAPK.^30^, ^31^ It is known that WSE causes macrophage dysfunction and increases metalloproteinase activity, including MMP‐2 and MMP‐9, leading to cell invasion and migration.^32^ Recently, Xu et al associated higher M1/M2‐macrophage infiltration, adenocarcinoma histology and never smokers with CD47 expression in NSCLC tumor.^33^ It is possible that in nonsmoker patients, the EGFR mutation contributes to CD47 overexpression and WSE causes alteration in macrophages avoiding tumor cell recognition leading to growing tumor cell and migration.

Shorter PFS was a feature of patients with EGFR‐mutant NSCLC having CD47 150+, possibly due to a cross‐talk between EGFR and CD47 signaling. It is known that CD47 is associated with other receptor tyrosine kinases (RTKs), for example, MET (39) and vascular endothelial growth factor receptor 2 (VEGFR2). VEGFR phosphorylation is inhibited when CD47 binds TSP1 expressed in endothelial and T cells.^13^ In different malignancies such as skin and breast cancer, relationship between EGFR mutations and CD47 is evidenced since EGFR inhibition exerts strong antitumor response through CD47 downregulation and vice versa.^34^ We hypothesize that CD47 modulates downstream EGFR signaling and it consequently affects response to EGFR‐TKI treatments since CD47 is associated with integrins and these molecules phosphorylate and activate several RTKs such as c‐MET, platelet‐derived growth factor receptor (PDGFR) and VEGFR.^35^, ^36^ Besides, in the tumor microenvironment, CD47 overexpression in cancer cells allows immune system evasion through inhibition of macrophage phagocytosis and regulation of T and NK cells. Similar to PD‐L1, upregulation through EGFR activation either by EGF ligand or oncogenic mutations, CD47 could be overexpressed as a result of PI3K‐AKT and MEK‐ERK axes upper activation in NSCLC cells.^28^ To the best of our knowledge, this is the first study that reports tumor CD47 overexpression associated with the presence of EGFR activating mutations and its negative impact on survival of NSCLC patients.

Finally, anti‐CD47 antibody‐based therapies are being developed to restore macrophage immunosurveillance, increasing immune recognition, thereby preventing tumor growth and metastasis.^11^ Currently, there are two therapeutic approaches targeting CD47 in clinical trials for hematologic and solid malignancies: (1) Hu5F9‐G4, an anti‐CD47 antibody tested alone or in combination with cetuximab (NCT02953782), rituximab (NCT02953509) or azacitidine (NCT03248479) in colorectal cancer, non‐Hodgkin's lymphoma and acute myeloid leukemia; and (2) TTI‐621, a fusion protein combining CD47 and the Fc region of IgG1 preventing delivery of anti‐phagocytic signals (NCT02663518). Moreover, antibody‐based fusion proteins have been designed targeting EGFR and CD47 with promising results.^37^ Further molecular and clinical studies are required to fully understand the interaction between CD47 and EGFR.

## 5 CONCLUSION

Overexpression of CD47 was not a prognostic factor for PFS and OS. Nevertheless, among patients with CD47 and EGFR (+), overexpression has a negative impact on clinical outcomes. This subset of patients is potentially eligible for combined CD47/EGFR therapies. However, further studies evaluating the mechanism between CD47 and EGFR are warranted.
