# Supplementary figures and images for: Association between CD47 expression, clinical characteristics and prognosis in patients with advanced non‐small cell lung cancer
Source: Cancer Med. 2020 Feb 11;9(7):2390–402. doi: 10.1002/cam4.2882 (PMC7131854; doi:10.1002/cam4.2882)

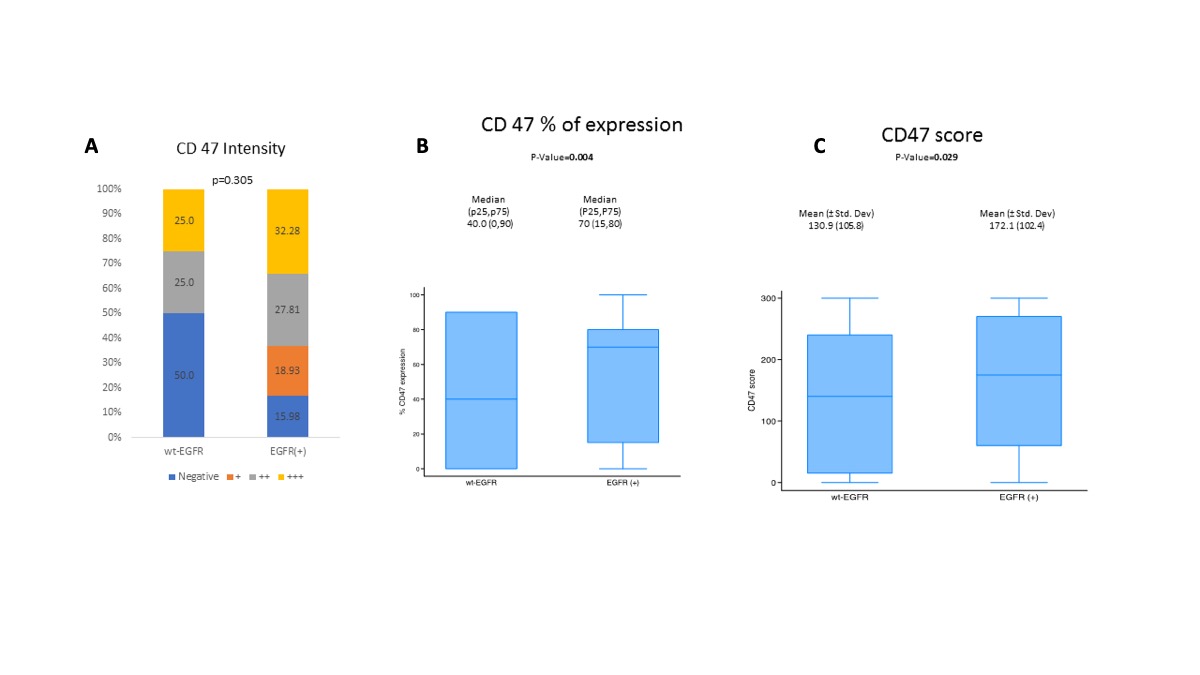

Supplement: Supplementary file 1 [file CAM4-9-2390-s001.jpg]
